# Calibration of miniature air quality detector monitoring data with PCA–RVM–NAR combination model

**DOI:** 10.1038/s41598-022-13531-4

**Published:** 2022-06-04

**Authors:** Bing Liu, Yirui Zhang

**Affiliations:** 1 Public Foundational Courses Department, Nanjing Vocational University of Industry Technology, Nanjing, 210023 China; 2 School of Intelligent Manufacturing, Sanmenxia Polytechnic, Sanmenxia, 472000 China

**Keywords:** Atmospheric science, Environmental sciences

## Abstract

The development of miniature air quality detectors makes it possible for humans to monitor air quality in real time and grid. However, the accuracy of measuring pollutants by miniature air quality detectors needs to be improved. In this paper, the PCA–RVM–NAR combined model is proposed to calibrate the measurement accuracy of the miniature air quality detector. First, correlation analysis is used to find out the main factors affecting pollutant concentrations. Second, principal component analysis is used to reduce the dimensionality of these main factors and extract their main information. Thirdly, taking the extracted principal components as independent variables and the observed values of pollutant concentrations as dependent variables, a PCA–RVM model is established by the relevance vector machine. Finally, the nonlinear autoregressive neural network is used to correct the error and finally complete the establishment of the PCA–RVM–NAR model. Root mean square error, goodness of fit, mean absolute error and relative mean absolute percent error are used to compare the calibration effect of PCA–RVM–NAR model and other commonly used models such as multiple linear regression model, support vector machine, multilayer perceptron neural network and nonlinear autoregressive models with exogenous input. The results show that, no matter which pollutant, the PCA–RVM–NAR model achieves better calibration results than other models in the four indicators. Using this model to correct the data of the miniature air quality detector can improve its accuracy by 77.8–93.9%.

## Introduction

Certain air pollutants, such as PM_2.5_, PM_10_, CO, NO_2_, SO_2_, O_3_ ("two dusts and four gases") can affect human health and cause respiratory diseases and cardiovascular diseases^1–3^. According to statistics, more than 3 million people die worldwide due to air quality problems every year^4,5^. Therefore, obtaining air pollutant concentration information is very necessary to control air pollution and prevent health problems caused by air pollution.

### Air quality monitoring platform

Many large cities in developed countries have established some air quality monitoring stations (national control points) in order to obtain information on the concentration of air pollutants. The concentrations of pollutants monitored by these air quality monitoring stations are relatively accurate. However, due to the high cost of establishing monitoring stations and high maintenance costs, the deployment of monitoring stations is relatively sparse. Another disadvantage of national control point monitoring is that the release of data is delayed, making it difficult to monitor the concentration of air pollutants in the entire region in real time. The development of miniature air quality detectors effectively overcomes these shortcomings of reference monitoring stations. The miniature air quality detector has low production and maintenance costs and is easy to install, so it can realize grid deployment and control of specific areas. For this specific areas where the miniature air quality detector is installed for the convenience of monitoring, this paper calls them self-built points. Another advantage of the miniature air quality detector is that it is easy to read the readings, so it can realize real-time monitoring of the concentration of air pollutants^6,7^. In addition, while monitoring the concentration of air pollutants, it can also monitor meteorological parameters such as temperature, humidity, wind speed, air pressure, and precipitation in the region.

Electrochemical sensors are one of the core components of many miniature air quality detectors. It works by reacting with the measured gas and producing an electrical signal proportional to the gas concentration. The gas reacts with the sensor through the tiny capillary-shaped openings and reaches the electrode surface, so that an appropriate amount of gas reacts with the sensing electrode to form a sufficient electrical signal, and finally achieve the purpose of monitoring. The miniature air quality detector will have zero drift or span drift after a period of use. In addition, unconventional pollutants in the air, weather factors, etc. will also cause errors in the measurement of the miniature air quality detector^8^. Therefore, it is very meaningful to establish a pollutant concentration prediction model to calibrate the self-built point data.

### Introduction to air quality prediction model

At present, many researchers have studied air quality prediction models. The main research methods are divided into two categories: chemical mechanism prediction and statistical model prediction. The chemical mechanism prediction is to quantitatively describe the changes of atmospheric pollutants in a certain area by using the numerical method of atmospheric dynamics and comprehensively considering the atmospheric physical and chemical mechanism^9,10^. Chemical mechanism prediction has the advantages of multi-scale and openness, but the main disadvantage is that the uncertainty of pollutant emission sources is large, the calculation time is long, and the prediction accuracy is not high. Statistical model forecasting first uses statistical methods to screen out meteorological factors that are strongly correlated with air pollution concentrations, and then uses statistical models to establish quantitative relationships between them and air pollution concentrations. Statistical model forecasting has the advantages of simplicity and economy, good forecast timeliness and accuracy, so it is widely used in air quality forecasting.

Traditional statistical forecasting models include Multiple Linear Regression (MLR) model^11–13^, grey models^14^, hidden Markov models^15,16^, time series models^17,18^ and so on. These traditional models are simple in structure, strong in interpretability and short in operation time, and are often used in air quality forecasting in recent years. Suriano et al. designed and developed the SentinAir system for field evaluation of sensor performance. In order to evaluate the system function and capability, indoor and outdoor experiments were performed independently. Linear regression (LR) and multiple linear regression models were used to calibrate the ten sensor data. The results show that the calibration effect of the MLR model is better than that of the LR model because it allows the quantification of the interfering effects of temperature, relative humidity and other gases^19^. However, the factors affecting air quality are complex, and it is difficult for these models to accurately reflect the nonlinear relationship between various factors and air quality. With the rise of big data and artificial intelligence, artificial neural networks^20–22^ have also been used to predict air quality. Arsic et al. used multiple regression analysis and artificial neural network to predict ground-level ozone concentrations in the close vicinity of the city of Zrenjanin (Serbia). The comparison results show that the artificial neural network has a better effect in monitoring the ozone concentration than the multiple linear regression model^23^.

Although the prediction effect of artificial neural network is good, neural network usually requires more data than traditional machine learning algorithms, and the output results are difficult to interpret. Random forest algorithm^24–26^ is also commonly used to predict air quality in recent years, but random forest is prone to overfitting in some noisy regression or classification problems. Support Vector Machine (SVM) can cleverly solve small sample, high-dimensional, nonlinear problems, and it follows the principle of structural risk minimization. Suarez Sanchez et al. used 2006–2008 experimental data on air pollutants to create a highly nonlinear model of the air quality in the Aviles urban nucleus (Spain) based on SVM techniques^27^. Liu et al. successfully predicted the concentration of air pollutants in Nanjing with the help of support vector regression machine, and calibrated the measurement data of the miniature air quality detector^28^.

However, for the air quality prediction problem, the support vector machine model also has certain shortcomings. First, as the dimension of training samples increases, the model prediction time is prolonged, which seriously restricts the timeliness of the model. Second, there are many parameters in the principle of the support vector regression machine^29,30^. In addition to the kernel function parameters, the penalty factor *C* and the radius of the insensitive loss area $\varepsilon$ will have a greater impact on the accuracy of the model, and it is difficult to establish a high-precision air quality prediction model. To address these issues, a Bayesian framework-based sparse probabilistic learning model relevance vector machine (RVM) is introduced in this paper to predict air quality. The relevance vector machine uses the active correlation decision theory to realize the sparseness of the model, which greatly reduces the amount of calculation, and the time of model prediction is better controlled. In addition, some model parameters can obtain the optimal solution through self-adaptive iteration, and there are few adjustment parameters, which is convenient for model optimization.

The main work of this paper is to find out the influencing factors affecting the concentration of six types of air pollutants through correlation analysis, and then use Principal Component Analysis (PCA) to extract the main information in these influencing factors. Then, these main information are used as input, the concentration of pollutants in the air is used as output, and the air quality prediction model is established with the help of relevance vector machine. Finally, the prediction residuals are corrected by the Nonlinear Autoregressive (NAR) neural network to further improve the prediction accuracy of the model. We call this combined model the PCA–RVM–NAR combined model. In practical applications, this model has achieved good results in air quality prediction, and it can provide a reference model for air quality prediction and data calibration of miniature air quality detectors.

## Material and methods

### Data source and preprocessing

The emergence and development of miniature air quality detectors provide the possibility for grid and real-time monitoring of air quality. However, its measurement is affected by many factors, so the measurement data will have errors. This paper uses a statistical model to calibrate it. A total of two sets of data (http://www.mcm.edu.cn/html_cn/node/b0ae8510b9ec0cc0deb2266d2de19ecb.html) are used in this study to establish the calibration model of the miniature air quality detectors. The first set of data comes from an air quality monitoring station in Nanjing, which contains 4200 sets of data and is considered accurate in this paper. It recorded the hourly concentration of two dusts and four gases from November 14, 2018 to June 11, 2019. The second set of data is provided by a miniature air quality detector juxtaposed with the air quality monitoring station. It contains 234,717 sets of data, and the interval between each set of data is no more than five minutes. The second set of data includes not only the concentration of two dusts and four gases, but also five meteorological parameters such as temperature, humidity, wind speed, air pressure, and precipitation.

Data preprocessing is the first step in establishing the data correction model of the miniature air quality detector. Data that is more than 3 times the mean value of the left and right nearest neighbors is regarded as an outlier and eliminated in this paper. Then average the measured values of the self-built point within an hour to compare with the data of the national control point, and delete some data that cannot correspond to the self-built point and the national control point. After data preprocessing, a total of 4135 sets of corresponding data are obtained, and Table 1 shows them.

**Table 1:** Descriptive statistics of pollutant concentrations and meteorological parameters measured by national control point and self-built point after pretreatment.

Input variable	Ranges	Mean	Standard deviation	Skewness	Kurtosis
PM_2.5_ (μg/m^3^)	1 to 216.883	64.127	37.328	0.988	0.701
PM_10_ (μg/m^3^)	2 to 443.25	102.391	65.267	1.476	2.862
CO (mg/m^3^)	0.05 to 3.895	0.863	0.452	1.463	3.136
NO_2_ (μg/m^3^)	0.947 to 157.136	45.209	28.403	0.653	− 0.259
SO_2_ (μg/m^3^)	1 to 651.3	19.397	18.723	12.781	342.11
O_3_ (μg/m^3^)	0.579 to 259	61.586	40.941	1.091	2.035
Wind speed (m/s)	0.133 to 2.387	0.7	0.346	0.862	0.748
Pressure (Pa)	996.871 to 1039.8	1018.8	8.889	− 0.093	− 0.599
Precipitation (mm/m^2^)	0 to 312.1	132.084	87.004	0.245	− 0.728
Temperature (°C)	− 3.882 to 37.944	11.882	8.603	0.625	− 0.399
Humidity (rh%)	10.667 to 100	68.903	21.931	− 0.487	− 0.756

### Data exploratory analysis

**Figure 1:**
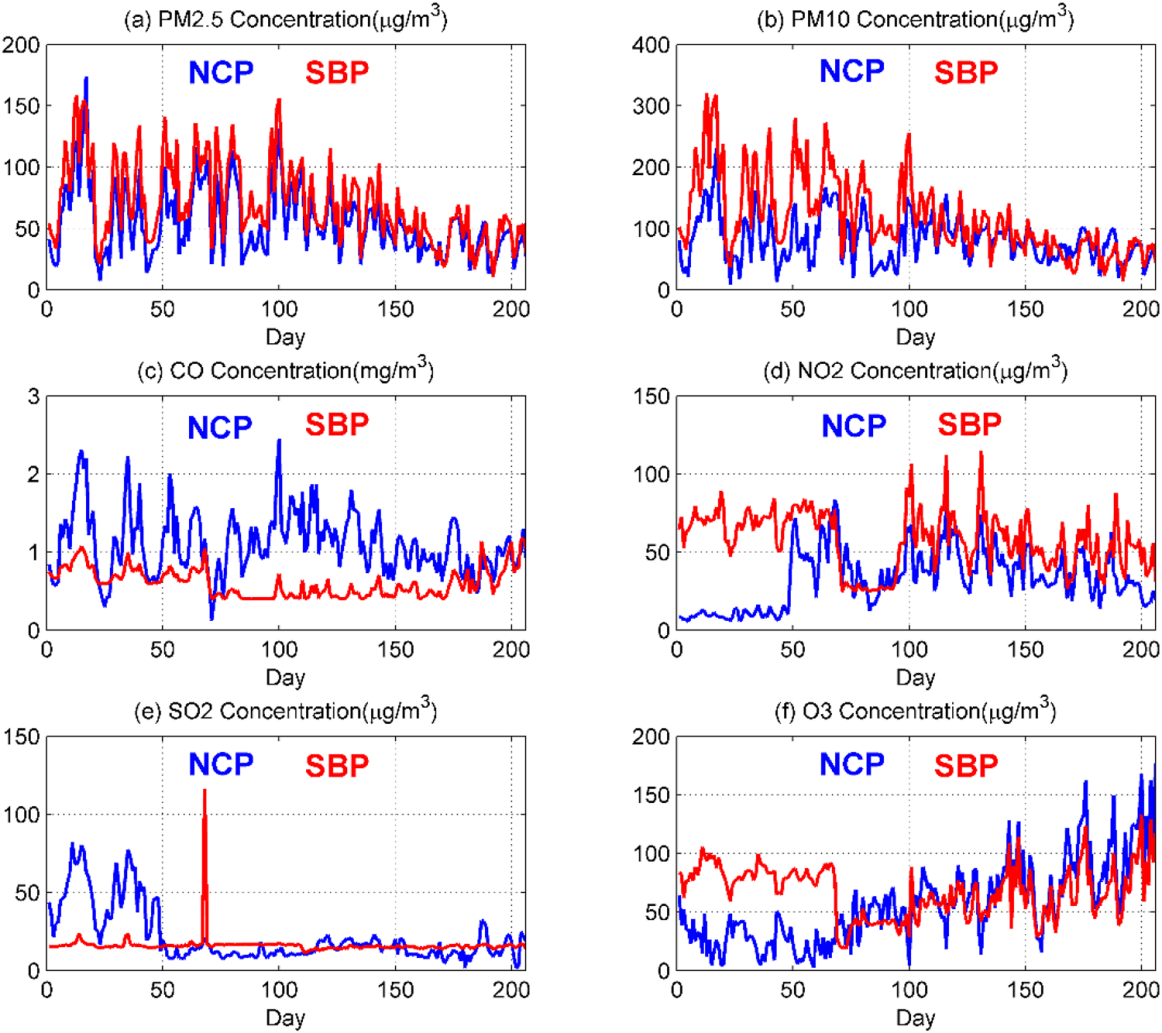
Comparison of daily average data of six types of pollutants at national control point (NCP) and self-built point (SBP). Figures are generated using Matlab (Version R2016a, https://www.mathworks.com/) [Software].

Exploratory analysis of data can give us a deeper understanding of the interrelationships between variables. In order to more intuitively reflect the relationship between the national control point measurement data and the self-built point measurement data, we average the measurement data on a daily basis and conduct visual analysis^6,9^. It can be seen from Fig. 1 that no matter what kind of pollutants it is, the general trend of the self-built point data and the national control point data is the same, but there are also certain errors. The difference between PM_2.5_ and PM_10_ is relatively small, indicating that the miniature air quality detector has high accuracy in measuring the concentrations of these two types of pollutants. The errors of NO_2_ and O_3_ in the previous period are relatively large, and the errors in the latter period are relatively small. It may be that the climate has a great influence on the concentration of these two pollutants measured by the miniature air quality detector. The measurement errors of CO and SO_2_ are large, indicating that it is difficult to monitor the concentrations of these two pollutants with a miniature air quality detector.

**Figure 2:**
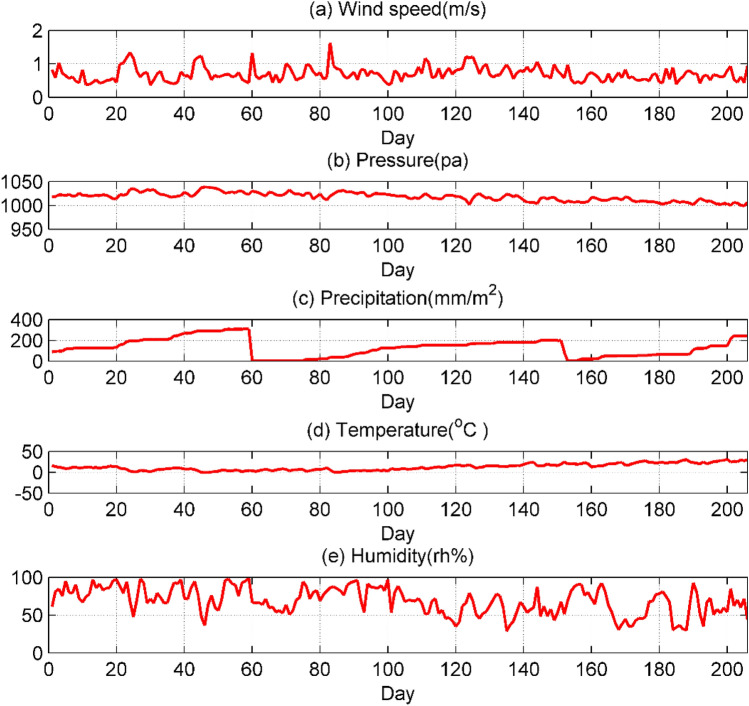
Variation of the daily average value of five meteorological parameters.

Figure 2 is a line chart of the changes of five meteorological parameters with time. It can be seen that there is abundant rain in this area, the daily average temperature is relatively mild, the daily average air pressure is stable between 1000–1050 Pa, and the air humidity and wind speed change more obviously. Further discussion and analysis are needed to find the relationship between meteorological parameters and the concentrations of the six types of pollutants.

**Figure 3:**
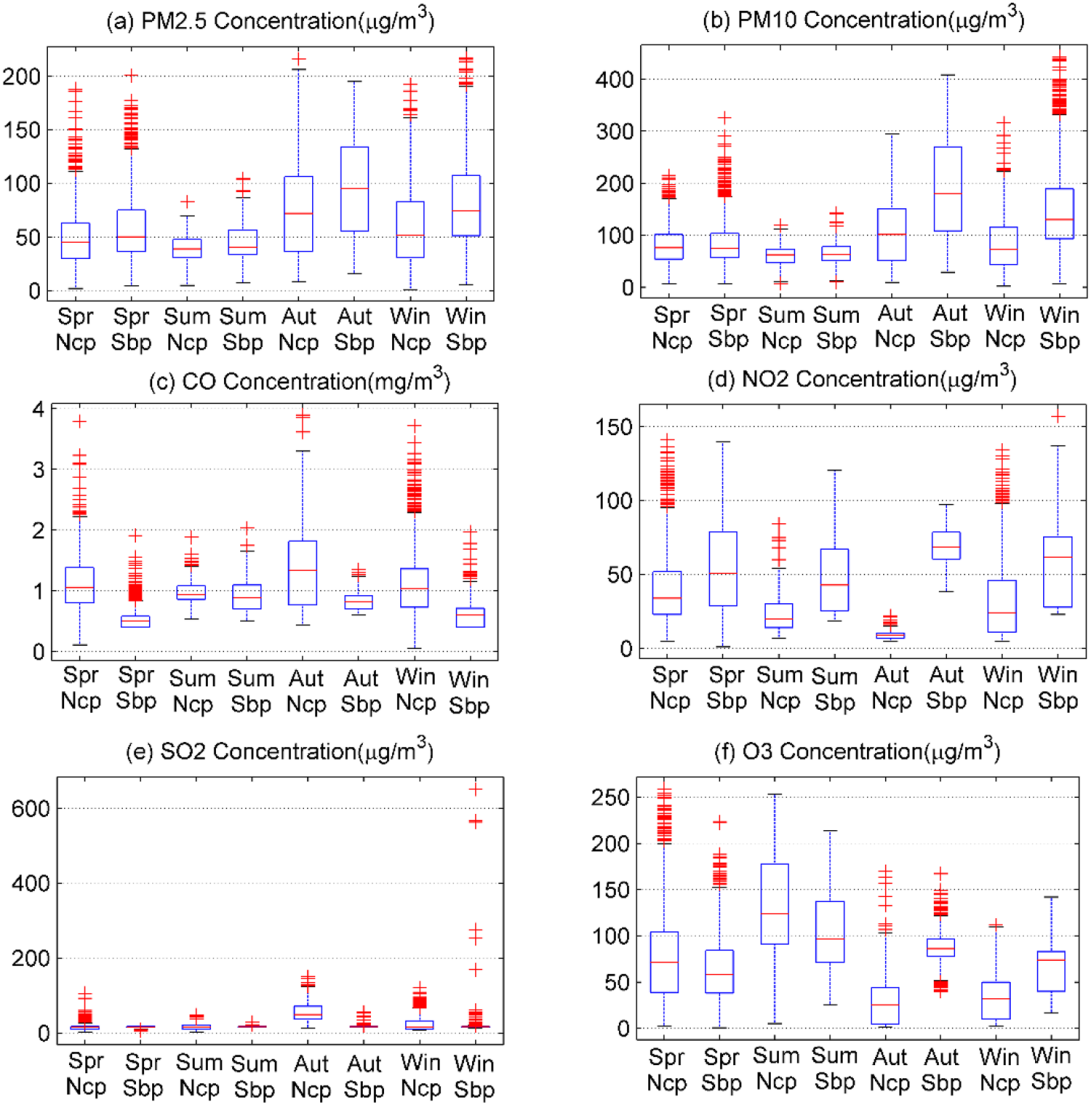
Comparing the concentration of six types of pollutants at national control sites (Ncp) and self-built sites (Sbp) on a seasonal basis.

The measurement error of the miniature air quality detector may have a certain relationship with the meteorological parameters, and there are obvious differences in the meteorological parameters in different seasons. We have drawn a boxplot^31^ of the six categories of pollutants by season as shown in Fig. 3. It can be seen that the concentrations of PM_2.5_, PM_10_, CO, and SO_2_ are the highest in autumn and winter. The main reason is that the temperature in autumn and winter is lower, and it is difficult for the lower air and upper air to generate convection, resulting in slower diffusion of pollutants. The reason for the high concentration of O_3_ in summer is the strong solar radiation and high temperature in summer, which is easy to cause photochemical smog and secondary ozone production. The slightly higher NO_2_ concentration in spring may be related to thunderstorms. In addition, the errors between the measured and actual values of the six types of pollutants have obvious differences in the four seasons, indicating that meteorological parameters will affect the measurement of the miniature air quality detector.

### Correlation analysis

The concentration of pollutants in the air is an important criterion for evaluating air quality. Different geographical environments have different influence factors on the concentration of air pollutants. The Pearson correlation coefficient is used in this paper to screen the main factors affecting air quality^25,32^. Equation (1) is its expression, where $x_{i}$ is the value of the first variable, $y_{i}$ is the value of the second variable, $\overline{x}$ is the mean of $x$, $\overline{y}$ is the mean of $y$, and $n$ represents the number of samples. The value range of the Pearson correlation coefficient is [− 1, 1], and the larger its absolute value, the stronger the correlation between the two variables.

It can be seen from Table 2 that under the premise of the significant level 0.05, except for NO_2_ concentration and temperature, the other variables are significantly correlated with each other. The positive correlation between PM_2.5_ concentration and PM_10_ concentration is the highest, and the correlation coefficient is 0.89, indicating that they have the same trend of change. The negative correlation between temperature and air pressure is the highest, and the correlation coefficient is − 0.85, indicating that there is a reverse trend between them.

1 $$ r = \frac{{\mathop \sum \nolimits_{i = 1}^{n} (x_{i} - \overline{x})(y_{i} - \overline{y})}}{{\sqrt {\mathop \sum \nolimits_{i = 1}^{n} (x_{i} - \overline{x})^{2} } \cdot \sqrt {\mathop \sum \nolimits_{i = 1}^{n} (y_{i} - \overline{y})^{2} } }} $$

**Table 2:** Pearson linear correlation coefficient between the concentrations of six types of air pollutants measured at national control point and five meteorological parameters measured at self-built point (Band * indicates significant correlation at a significant level of 0.05).

Variable	PM_2.5_	PM_10_	CO	NO_2_	SO_2_	O_3_	Wind speed	Pressure	Precipitation	Temperature	Humidity
PM_2.5_	1.00	0.89*	0.66*	0.26*	0.29*	− 0.26*	− 0.23*	0.89*	− 0.70*	− 0.16*	0.18*
PM_10_		1.00	0.63*	0.34*	0.35*	− 0.19*	− 0.18*	0.38*	− 0.10*	− 0.03*	− 0.09*
CO			1.00	0.30*	0.31*	− 0.27*	− 0.31*	− 0.07*	0.08*	− 0.05*	0.22*
NO_2_				1.00	− 0.34*	− 0.26*	− 0.36*	− 0.10*	− 0.14*	− 0.02	− 0.11*
SO_2_					1.00	− 0.28*	− 0.19*	0.19*	0.27*	− 0.10*	0.11*
O_3_						1.00	0.39*	− 0.45*	− 0.12*	0.68*	− 0.62*
Wind speed							1.00	0.09*	0.06*	0.07*	− 0.32*
Pressure								1.00	0.23*	− 0.85*	0.15*
Precipitation									1.00	− 0.14*	0.86*
Temperature										1.00	− 0.49*
Humidity											1.00

## Establishment of sensor calibration model

### Introduction to basic principles

The relevance vector machine is a sparse probability model similar to the support vector machine proposed by Tipping in 2000. It is a new supervised learning method. The model combines theories such as Markov's, Bayes's principle and maximum likelihood. Due to the high sparsity of the algorithm and the structure based on probabilistic learning, RVM can enable us to obtain high prediction accuracy. In addition, compared with the support vector machine, it greatly reduces the number of kernel functions involved in the prediction calculation and reduces the prediction calculation time. RVM also has the advantages of probabilistic prediction, automatic parameter setting and arbitrary use of kernel functions^33–35^.

2 $$ t_{n} = y(x_{n} ;\;\omega ) = \mathop \sum \limits_{n = 1}^{N} \omega_{n} k(x,\;x_{n} ) + \varepsilon_{n} $$

3 $$ p\left( {\left. {t_{n} } \right|x_{n} } \right) = N\left( {\left. {t_{n} } \right|y(x_{n} ),\;\sigma^{2} } \right) $$

4 $$ p\left( {\left. t \right|\omega ,\;\sigma^{2} } \right) = \left( {2\pi \sigma^{2} } \right)^{{ - \frac{N}{2}}} \exp \left\{ { - \frac{1}{{2\sigma^{2} }}\left\| {t - \Phi \omega } \right\|^{2} } \right\} $$

5 $$ p\left( {\left. \omega \right|\alpha } \right) = \mathop \prod \limits_{n = 0}^{N} N\left( {\left. {\omega_{n} } \right|0,\;a_{n}^{ - 1} } \right) $$

6 $$ p\left( {\left. {t_{*} } \right|t} \right) = \int p\left( {\left. {t_{*} } \right|\omega ,\;\alpha ,\;\sigma^{2} } \right)p\left( {\left. {\omega ,\;\alpha ,\;\sigma^{2} } \right|t} \right) \times d\omega d\alpha d\sigma^{2} $$

The relevance vector machine is not constrained by the Mercer condition when selecting the kernel function, it can achieve binary classification and probability output, and the running speed is fast. Let the training data samples be $\left\{ {x_{n} ,\;\left. {t_{n} } \right|n = 1,\;2, \ldots ,\;N} \right\}$, where $x_{n}$ is the input value,${ }t_{n}$ is the output value, $N$ is the number of data samples, Eq. (2) is the expression of the regression model, where $k(x,\;x_{n} )$ is the kernel function, $\omega = \left\{ {\omega_{n} } \right\}_{n = 0}^{N}$ is the weight value of each input quantity, $\varepsilon_{n}$ is the data noise and obeys the Gaussian distribution, $\varepsilon_{n} \sim N(0,\;\sigma^{2} )$, $\sigma^{2}$ is an unknown quantity. Thus, the Eq. (3) that satisfies the Gaussian distribution is obtained, where $t_{n}$ is related to $y(x_{n} )$ and $\sigma^{2}$, and $t_{n}$ is independent of each other. Equation (4) is the likelihood function of the training sample set, where $t = \{ t_{1} ,\;t_{2} , \ldots ,\;t_{N} \}^{T}$,${ }\omega = \{ \omega_{0} ,\;\omega_{1} , \ldots ,\;\omega_{N} \}^{T}$,$\Phi = \left[ {\varphi \left( {x_{1} } \right),\;\varphi \left( {x_{2} } \right), \ldots ,\;\varphi \left( {x_{N} } \right)} \right]^{T}$ is an $N \times \left( {N + 1} \right)$ matrix, and the expression of each column in the matrix is $\varphi (x_{n} ) = [1,\;k(x_{n} ,\;x_{1} ), \;k(x_{n} ,\;x_{2} ), \ldots ,\;k(x_{n} ,\;x_{N} )]^{T}$. The hyperparameter $\alpha = \{ \alpha_{0} ,\;\alpha_{1} , \ldots ,\;\alpha_{N} \}^{T}$ is introduced to solve $\omega$ and $\sigma^{2}$ in Eq. (4), $\omega_{n}$ satisfies the Gaussian distribution, and its expression is Eq. (5). Equation (6) is the expression of the input value $x_{*}$ and the output value $t_{*}$ of the prediction data set. According to the Bayesian and Markov properties and Eq. (6), Eq. (7) can be obtained by simultaneous simplification, where Eqs. (8)–(10) represent the covariance and weight mean.

7 $$ p\left( {\left. \omega \right|t,\;\alpha ,\;\sigma^{2} } \right) = (2\pi )^{{ - \frac{N + 1}{2}}} \left| {\Sigma } \right|^{{ - \frac{1}{2}}} \exp \left\{ { - \frac{1}{2}(\omega - \mu )^{T} {\Sigma }^{ - 1} (\omega - \mu )} \right\} $$

8 $$ {\Sigma } = (\sigma^{2} \Phi^{T} \Phi + A)^{ - 1} $$

9 $$ \mu = \sigma^{ - 2} \sum \Phi^{T} t $$

10 $$ A = diag\left( {a_{0} ,\;a_{1} , \ldots ,\;a_{N} } \right) $$

Equation (11) can be obtained after the maximum likelihood function is simplified. Find the partial derivative of $\alpha$ and $\sigma^{2}$ in Eq. (11), and let them be 0 to establish two equations. After simplification, Eqs. (12)–(13) can be obtained, where $\gamma_{n} = 1 - \alpha_{n} {\Sigma }_{nn}$, ${\Sigma }_{nn}$ is the element of row *n* and column *n* of ${\Sigma }$. $\alpha$ and $\sigma^{2}$ are obtained through the update iteration of Eqs. (12)–(13). At the same time, the weight posterior mean $\mu$ and the covariance matrix ${\Sigma }$ change continuously until the convergence condition or the maximum number of iterations is satisfied. In the iterative process, new optimal solutions $\alpha_{MP}$ and $\sigma_{MP}^{2}$ will be obtained, and most of the weights will approach 0, and the corresponding basis functions will be ignored, which reflects the sparsity of the RVM model, and other weights will approach a constant, and the corresponding basis functions are called relevance vectors. The expected value $y_{*}$ and the noise variance $\sigma^{2}$ (Eqs. (14)–(15)) can be obtained by predicting the relationship between the input value $x_{*}$ and the output value $t_{*}$ of the data set (Eq. (6)), where $x_{*}$ is the sample to be predicted, $y_{*}$ is the mean of the output value $t_{*}$.

11 $$ p(\left. t \right|\alpha ,\;\sigma^{2} ) = \int p(\left. t \right|\omega ,\;\sigma^{2} )p(\left. \omega \right|\alpha )d\omega = (2\pi )^{{ - \frac{N}{2}}} \left| {\Sigma } \right|^{{ - \frac{1}{2}}} \exp \left[ {\frac{1}{2} \times (\omega - \mu )^{T} {\Sigma }^{ - 1} (\omega - \mu )} \right] $$

12 $$ \alpha_{n}^{new} = \frac{{\gamma_{n} }}{{\mu_{n}^{2} }} $$

13 $$ (\sigma^{2} )^{new} = \frac{{\left\| {t - \Phi \mu } \right\|^{2} }}{{N - \mathop \sum \nolimits_{n}^{N} \gamma_{n} }} $$

14 $$ y_{*} = \mu^{T} \varphi (x_{*} ) $$

15 $$ \sigma_{*}^{2} = \sigma_{MP}^{2} + \varphi (x_{*} )^{T} {\Sigma }\varphi (x_{*} ) $$

### PCA–RVM model construction

Air quality is affected by a variety of factors, and the relationship between the influencing factors is intricate. The variables input to the model have a great relationship with the accuracy of prediction. According to the previous correlation analysis, it can be seen that the pollutant concentration measured by the miniature air quality detector and the five meteorological parameters are significantly related to the air quality, so they all have a certain impact on the air quality. In addition, since the input variables also affect each other, if all variables are directly input into the relevance vector machine, some repetitive information will be input into the model, which not only makes the training time of the model longer, but also makes the model generalization ability deteriorates.

Principal component analysis is a method of data dimensionality reduction and denoising. It converts a series of components that are originally related in the system into several uncorrelated components through orthogonal transformation, and this group of components after conversion is called the principal component. Then, according to the contribution of each component to the data system, the principal components are recombined to highlight the hidden features in the original data to construct a mapping matrix, and then the original data is transformed by the mapping matrix to achieve the purpose of denoising^28^. The process of principal component analysis is generally as follows: (i) Standardize the original data; (ii) Calculate the correlation coefficient matrix R; (iii) Calculate the eigenvalues and eigenvectors; (iv) Select $p$ $(p \le m)$ principal components and calculate the comprehensive evaluation value. In this paper, the principle of extracting the number of principal components is that the cumulative contribution rate exceeds 99%.

**Figure 4:**
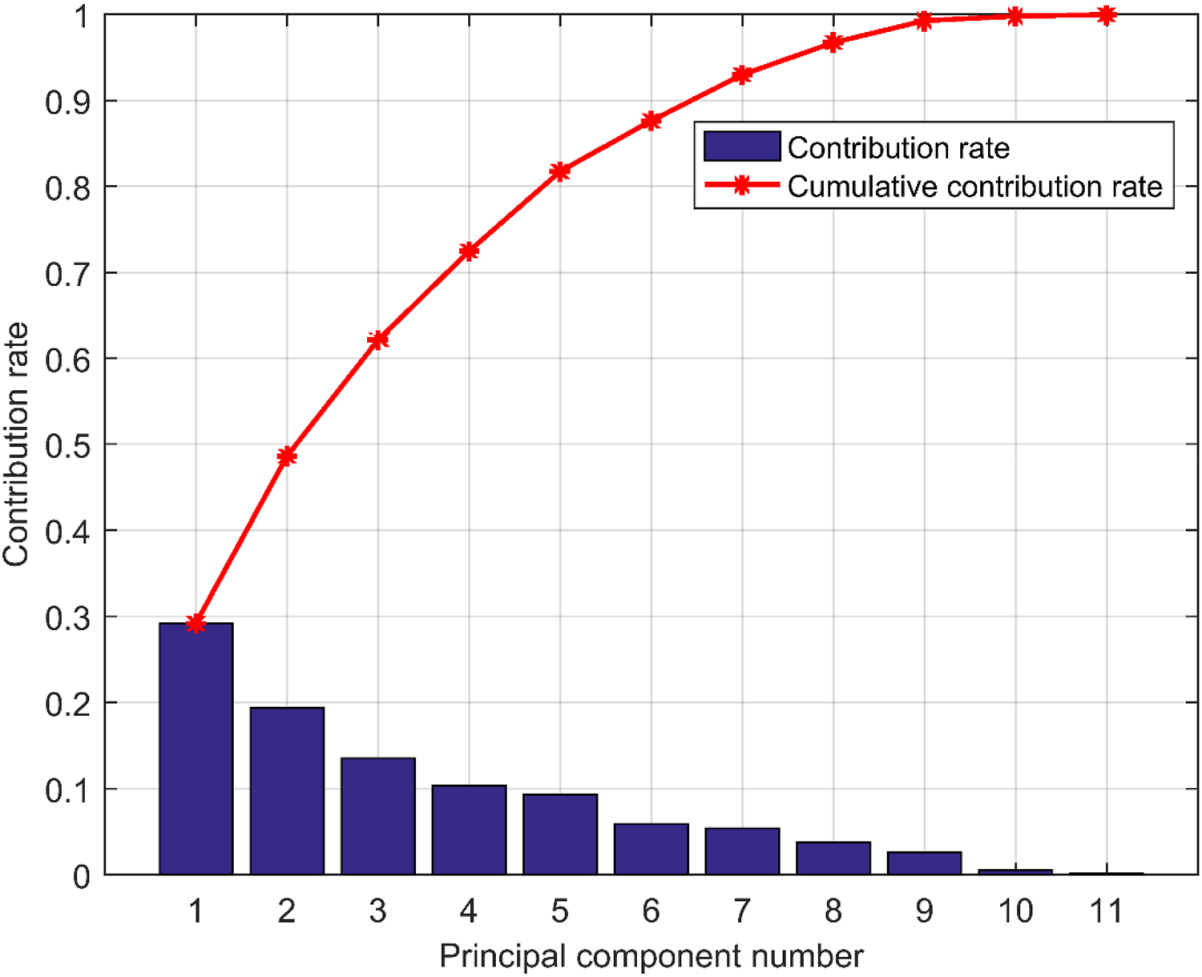
The principal component contribution rate and the cumulative contribution rate of the self-built point measurement data.

Figure 4 shows the principal component contribution rate and the principal component cumulative contribution rate after dimension reduction by principal component analysis. It can be seen that the contribution rate of the first principal component reaches 29.2%, and the contribution rate of the second, third and fourth principal components also exceeds 10% respectively, and the cumulative contribution rate of the first four principal components exceeds 70%. In addition, it can be seen from the broken line in the figure that the cumulative contribution rate of the first 9 principal components has exceeded 99%, which is in line with the principle of the number of extracted principal components. It shows that PCA is effective for dimensionality reduction of air quality data, and can provide more reliable input for subsequent prediction.

After the principal component dimension reduction is performed on the original data, the first 9 principal components after dimension reduction are used as input independent variables, and the predicted values of six types of pollutant concentrations are used as output variables, and the relevance vector machine is used to build the air quality prediction model. This combined model is called the PCA–RVM model in this paper. Since the construction process of the six types of pollutant prediction models is similar, we take PM_2.5_ concentration as an example, and other pollutant concentration prediction models can be obtained similarly.

We randomly divided 4135 groups of data, 3000 groups are selected as the training set, and the other 1135 groups are selected as the test set, and used Matlab2016a for modeling. For the training of the RVM model, according to the RVM regression principle, it can be seen that the hyperparameter $\alpha$ and the noise $\sigma^{2}$ are not sensitive to the initial value, and the optimal value can be obtained by iterative adaptation. The kernel function of the relevance vector machine uses the Gaussian kernel function, because the Gaussian kernel function can obtain a very smooth estimation^36,37^. The value of the model kernel function width $\gamma$ is obtained by the grid optimization method, the optimization interval is [0.5, 10], and the step size is 0.5. Equation (16) is the expression of Root Mean Square Error (RMSE), where $y_{i}$ represents the target value, $w_{i}$ represents the model predicted value. In this paper, the RMSE between the target value of the sample training set and the model predicted value is used as the objective function for optimization. During the training process, for each parameter value, we train the model 10 times, and average the output values of the 10 training times as the final output of the model. Through empirical analysis, when γ = 1.5 is the optimal value, the PCA–RVM air quality prediction model is established.

16 $$ RMSE = \sqrt {\frac{1}{n}\mathop \sum \limits_{i = 1}^{n} (y_{i} - w_{i} )^{2} } { } $$

### PCA–RVM–NAR model construction

**Figure 5:**
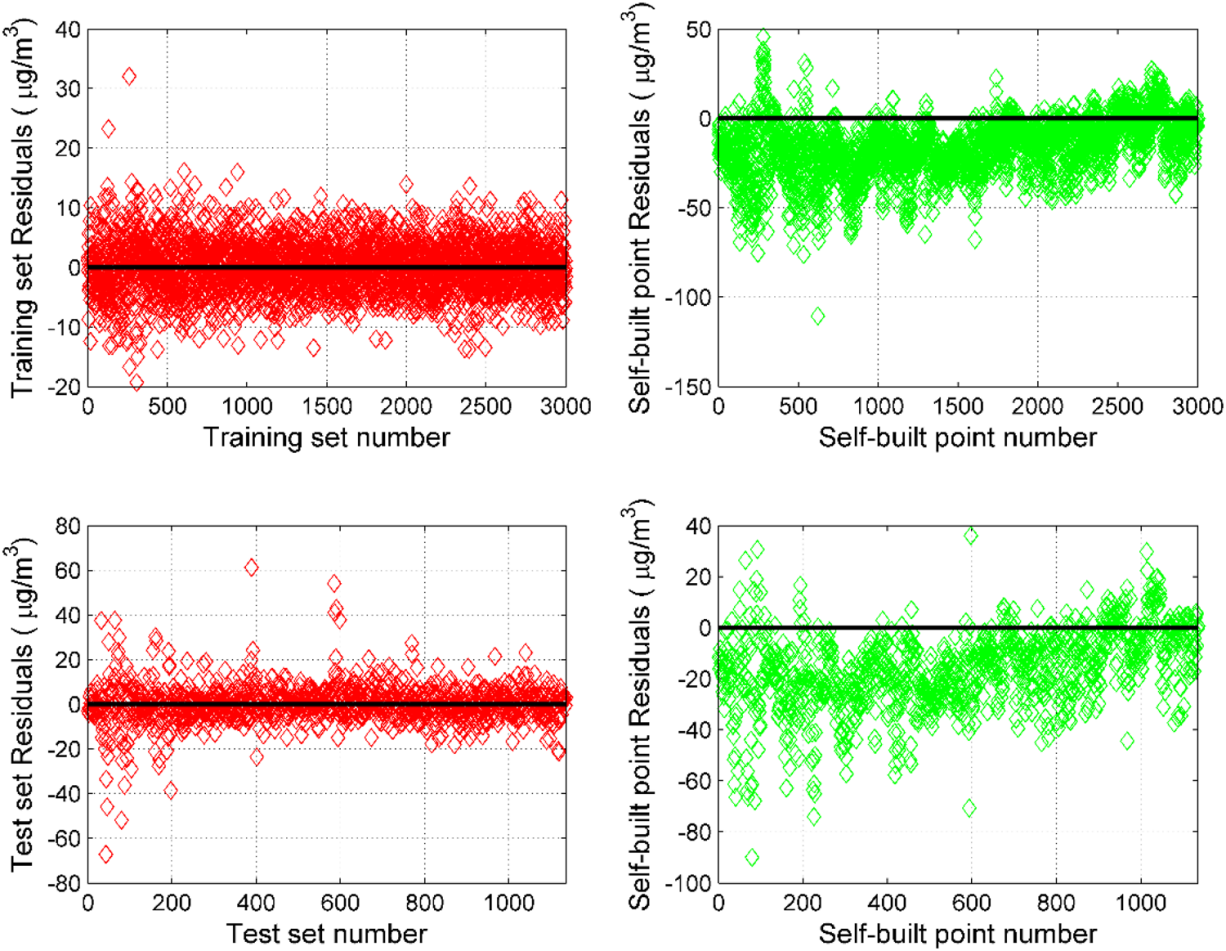
Comparison of PM_2.5_'s PCA–RVM model residuals and self-built point residuals. The comparison of the training set is on the left, and the comparison of the test set is on the right.

The PCA–RVM model can be used to calibrate the miniature air quality detector data. It can be seen from Fig. 5 that the residual of the PM_2.5_'s PCA–RVM model is greatly improved compared to the residual of the self-built point, whether it is the training set or the test set. In the training set, the residual of the model is concentrated in [− 10, 10], and the absolute value of the maximum residual is 32.06 μg/m^3^, while the residual of the self-built point is concentrated in [− 40, 20], and the absolute value of the maximum residual is 110.44 μg/m^3^. In the test set, the residual of the model is concentrated in [− 20, 20], and the absolute value of the maximum residual is 67.2 μg/m^3^, while the residual of the self-built point is concentrated in [− 50, 25], and the absolute value of the maximum residual is 90 μg/m^3^. The PCA–RVM model performs well in both the training set and the test set, indicating that the generalization ability of the model is good.

Although the PM_2.5_ concentration prediction effect of the PCA–RVM model is good, a set of time series residual data is obtained, and some residuals in the model are still high. Autoregressive integrated moving average model and NAR neural network model are commonly used to deal with time series data. This paper uses a NAR neural network to further mine the residual information.

**Figure 6:**
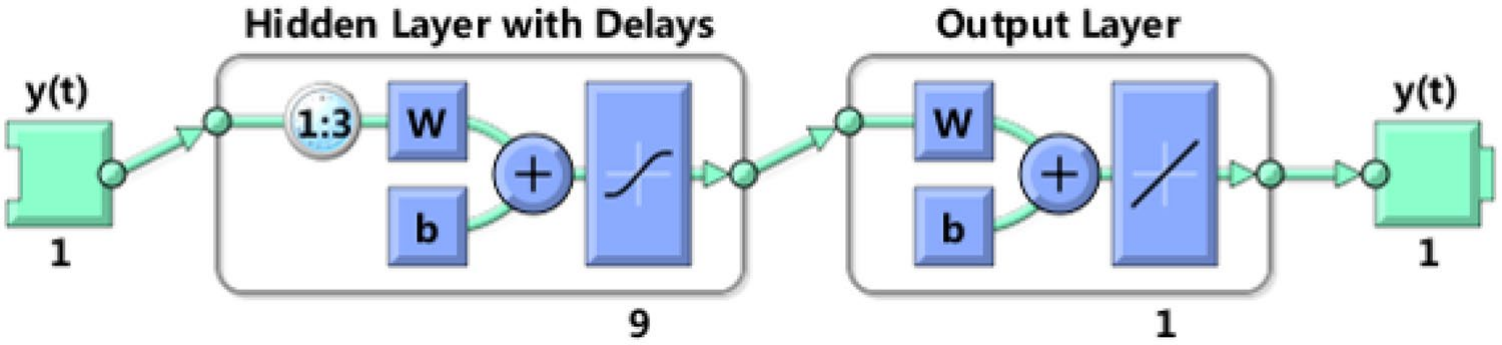
The frame structure of the PCA–RVM–NAR model, where the input is the residual of the PCA–RVM model. This network has 1 inputs, 1 hidden layer with 9 hidden neurons, 3 input delay orders, and 1 linear output layer leading to 1 output.

The NAR neural network belongs to the dynamic neural network and can be expressed by Eq. (17), where *y*(*t*) is the output value at the current moment, $y(t - 1),\;y(t - 2), \ldots ,\;y(t - d)$ are the output value at the historical moment, and $d$ is the delay order. NAR neural network consists of input layer, hidden layer and output layer^38^. For the selection of the number of neurons in the hidden layer and the order of input delay, we also use grid optimization to optimize in [5, 15] × [1, 5]. The training function of the NAR neural network adopts the default Levenberg–Marquardt (LM) algorithm in the Neural Net Time Series in Matlab. The core idea of the LM algorithm is to use the Jacobian matrix to replace the solution of the positive definite matrix in the gradient learning algorithm to optimize the operation efficiency of the training network. For the objective function, RMSE is also chosen as the objective function, and the final output is also obtained by averaging 10 times of training. After optimization, it is found that the optimal value is when the number of neurons in the hidden layer is 9 and the delay order is 3. The structure of the NAR neural network is shown in Fig. 6, where ${\text{w}}$ is the weight of the neural network model, and ${\text{b}}$ is the threshold of the neural network model. The PCA–RVM–NAR air quality prediction model has now been constructed.

17 $$ y(t) = f(y(t - 1),\;y(t - 2), \ldots ,\;y(t - d)) $$

**Figure 7:**
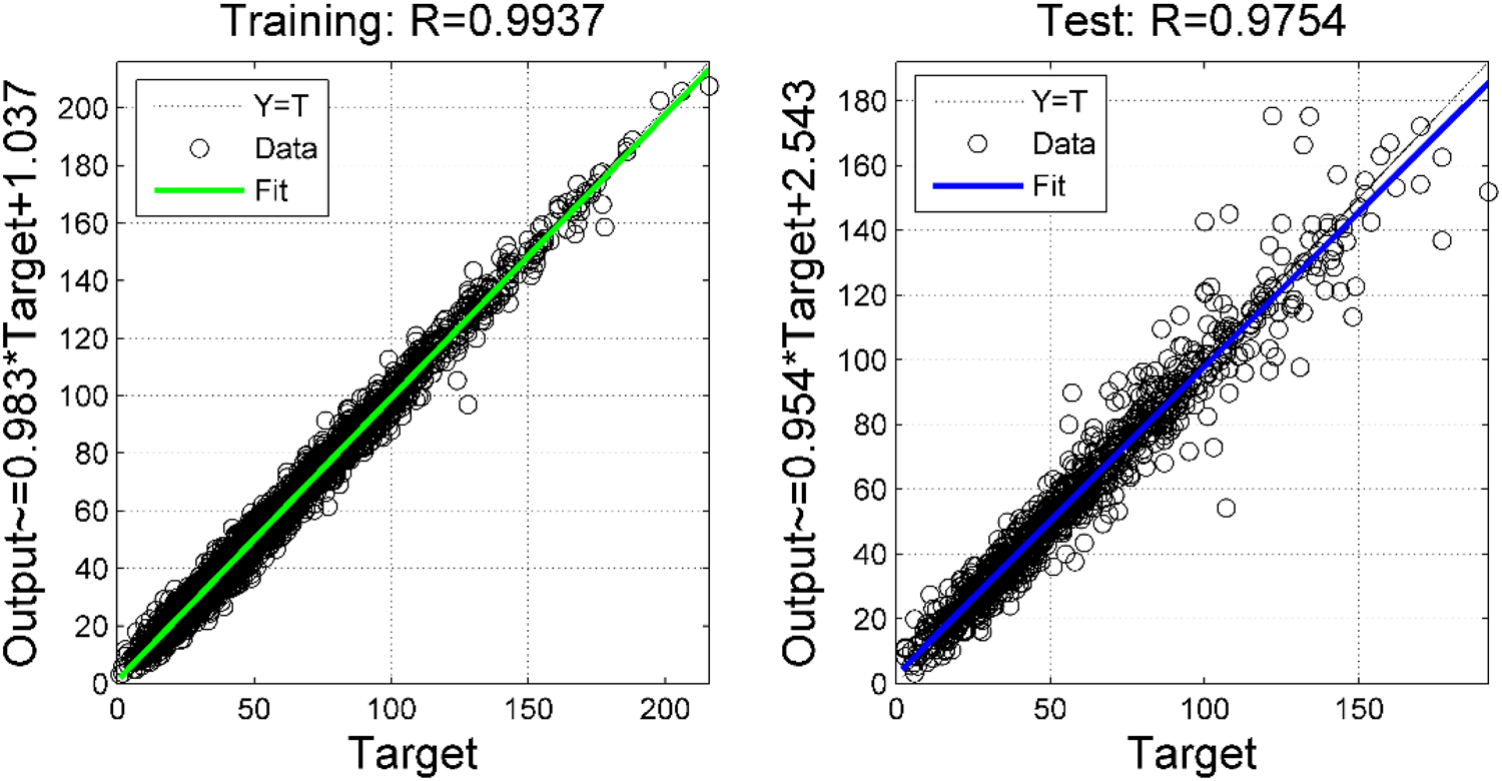
The prediction effect of PM_2.5_'s PCA–RVM–NAR model on the training set and test set.

Figure 7 shows the measured value of PM_2.5_ concentration at the national control point and the predicted value of PCA–RVM–NAR combined model. It can be seen that the change trend of the two is consistent, and the correlation coefficient between the measured value of the national control point and the predicted value of the PCA–RVM–NAR model is greater than 0.95 in both the training set and the test set. Both models in the training set and the test set passed the significance test at the significance level of 0.01. The regression coefficients in the two regression models are also close to 1, indicating that the PCA–RVM–NAR model is more accurate in PM_2.5_ concentration prediction.

**Figure 8:**
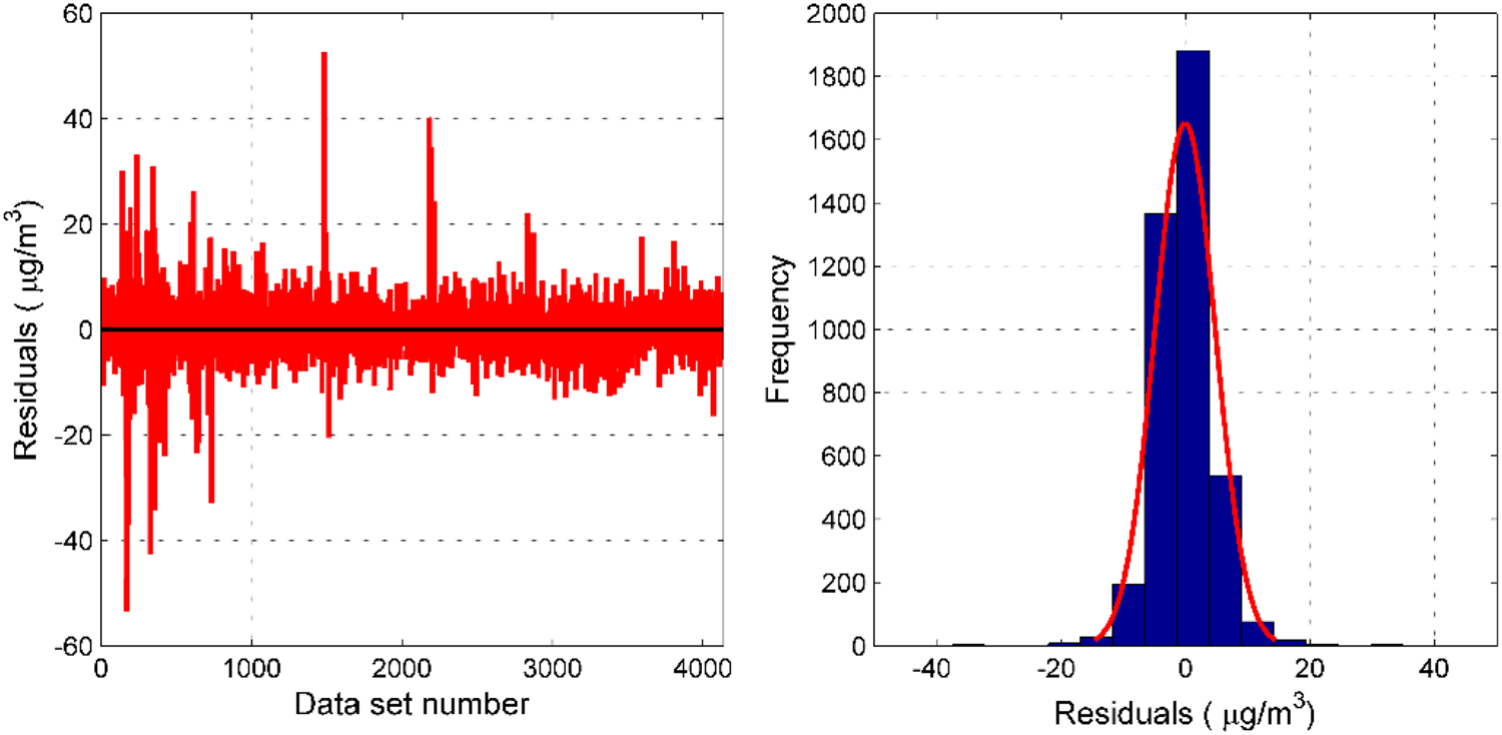
Residual test of PM_2.5_'s PCA–RVM–NAR model. The residual plot of the PCA–RVM–NAR model is seen on the left. The histogram of the residuals is seen on the right.

Residual analysis is also a necessary step in statistical modeling^12,15^. It can be seen from the residual analysis diagram in Fig. 8 that most of the residuals of the PCA–RVM–NAR model are concentrated in [− 10, 10], and the residuals are evenly distributed near the zero point. The absolute values of residuals at the 172nd and 1481st sample points are larger than 50 μg/m^3^. We checked the corresponding data, and the PM_2.5_ concentration measured at the national control point has changed greatly at this moment, indicating that the measurement residual of the model will increase when the pollutant concentration changes rapidly. In order to better display the residual characteristics of the model, this paper deletes these two points and draws a residual histogram. From the histogram we can see that the residuals are roughly normally distributed. A total of 3981 sets of data residuals are located in [− 10,10], exceeding 96.2%, and only 27 sets of residuals whose absolute value exceeds 20, do not exceed 0.5% of the total. In addition, 91.3% of the data prediction residuals are within 20%, and 73.3% of the data prediction residuals are within 10%.

## Discussion

The PCA–RVM–NAR combination model can calibrate the PM_2.5_ measurement concentration of the miniature air quality detector, and has achieved good results. In addition, multiple linear regression model, Support Vector Regression machine (SVR), Multilayer Perceptron neural networks (MLP) and Nonlinear Autoregressive models with Exogenous Inputs (NARX) can also calibrate the PM_2.5_ measurement concentration of the miniature air quality detector^39–41^. In order to visually compare the calibration effects of various models, Taylor diagram is used in this paper to compare them.

**Figure 9:**
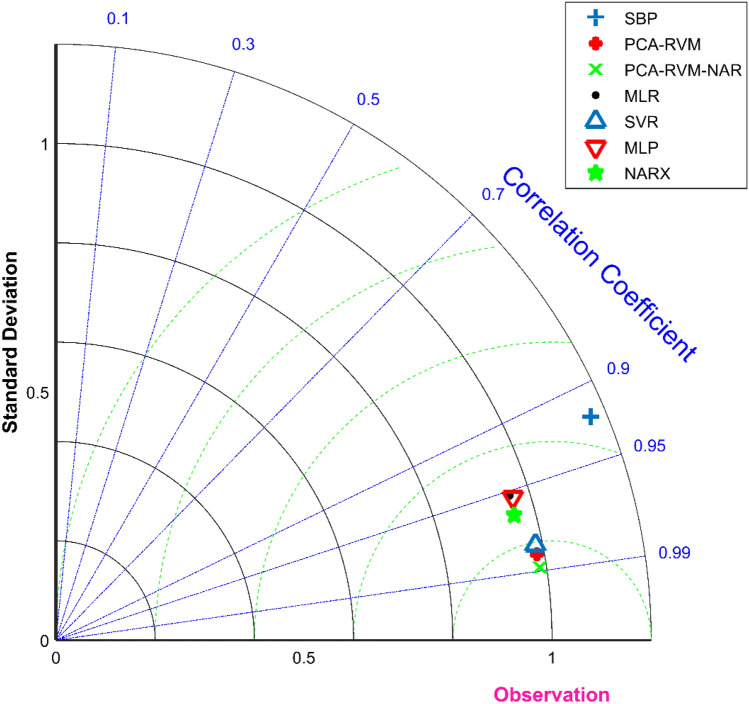
Taylor diagrams of the predicted PM_2.5_ concentration for the six models and the measured value of the self-built point, where SBP represents the self-built point.

Taylor diagram is a visual chart that can simultaneously represent three indicators of correlation coefficient, standard deviation and centered root mean square difference. The scatter points in the Taylor diagram represent different models, the radial line represents the correlation coefficient, the horizontal and vertical axes represent the standard deviation, and the dashed line represents the centered root mean square difference. Equation (1), Eqs. (18)–(19) are their expressions, where $y_{i}$ represents the true value, $w_{i}$ represents the model predicted value, $\overline{y}$ represents the mean value of $y$, and $\overline{w}$ represents the mean value of $w$. Taylor diagram can compare the relationship between model indicators from multiple perspectives and dimensions. It can be seen from Fig. 9 that the distance between the self-built point and the observation point (national control point) is the farthest, indicating that the PM_2.5_ measurement accuracy of the self-built point is the lowest, and the measurement value of the self-built point needs to be calibrated. Multiple linear regression model, multilayer perceptron neural network and NARX neural network can calibrate the PM_2.5_ measurement accuracy of self-built point, but the calibration accuracy needs to be improved. The calibration effect of the support vector machine and the PCA–RVM model is better, but in general, the PCA–RVM–NAR combined model given in this paper performs the best in the calibration of PM_2.5_ measurement accuracy.

18 $$ \sigma = \sqrt {\frac{1}{n}\mathop \sum \limits_{i = 1}^{n} (w_{i} - \overline{w})^{2} } $$

19 $$ E^{\prime} = \sqrt {\frac{1}{n}\mathop \sum \limits_{i = 1}^{n} [(y_{i} - \overline{y}) - (w_{i} - \overline{w})]^{2} } $$

In order to comprehensively compare the accuracy of the PCA–RVM–NAR model with other commonly used air quality prediction models, four commonly used indicators are used to compare the models in this paper^32,39^. These four indicators include Root Mean Square Error, Goodness of fit (R^2^), Mean Absolute Error (MAE) and relative Mean Absolute Percent Error (MAPE). Equation (16), Eqs. (20)–(22) are their expressions, where $y_{i}$ represents the measured values of six types of pollutants in the national control point, and $w_{i}$ represents the predicted values of various prediction models. The comparison of each indicator of two dusts and four gases is shown in Tables 3, 4, 5 and 6. It can be seen that the error of the self-built point is not only the largest in the PM_2.5_ measurement concentration, but also the largest in other pollutants. It should be noted that the R^2^ of some pollutants is negative, which is caused by the large measurement error of the self-built point. This indicator is eliminated when the calculation model improves the measurement accuracy. The support vector regression machine is obviously better than the MLR, MLP and NARX models in each evaluation index value, which shows that the SVR is more suitable for the calibration of the monitoring data of the miniature air quality detector. The performance of correlation vector machine is better than that of support vector regression machine in each evaluation indicator, and the PCA–RVM–NAR model proposed in this paper has the best performance in four indicators of six pollutants. The PCA–RVM–NAR model has the lowest improvement in the measurement accuracy of the miniature air quality detector is the RMSE of PM_2.5_. The measurement accuracy of this detector improves of the 77.8% considering the self-built point (RMSE = 22.436) and the PCA–RVM–NAR model (RMSE = 4.97). The PCA–RVM–NAR model has the highest improvement in the measurement accuracy of the miniature air quality detector is the MAPE of NO_2_. The measurement accuracy of this detector improves of the 93.9% considering the self-built point (MAPE = 2.129) and the PCA–RVM–NAR model (MAPE = 0.13).

20 $$ R^{2} = 1 - \frac{{\mathop \sum \nolimits_{i = 1}^{n} (y_{i} - w_{i} )^{2} }}{{\mathop \sum \nolimits_{i = 1}^{n} (y_{i} - \overline{y})^{2} }} $$

21 $$ MAE = \frac{1}{n}\mathop \sum \limits_{i = 1}^{n} \left| {y_{i} - w_{i} } \right| $$

22 $$ MAPE = \frac{1}{n}\mathop \sum \limits_{i = 1}^{n} \left| {\frac{{y_{i} - w_{i} }}{{y_{i} }}} \right| $$

**Table 3:** The RMSE of self-built point and various air quality prediction models, in which national control point is used as comparison object.

Input variable	Self-built point	PCA–RVM	PCA–RVM–NAR	MLR	SVR	MLP	NARX
PM_2.5_	22.436	5.873	4.970	10.149	8.649	10.777	8.800
PM_10_	66.263	10.605	7.740	20.050	11.656	19.126	13.911
CO	0.679	0.131	0.085	0.344	0.175	0.304	0.158
NO_2_	37.183	6.597	5.049	16.653	7.725	13.216	8.081
SO_2_	26.24	4.018	2.843	15.305	4.116	9.984	5.104
O_3_	45.673	8.669	6.627	21.451	11.304	18.603	12.477

**Table 4:** The R^2^ of self-built point and various air quality prediction models, in which national control point is used as comparison object.

Input variable	Self-built point	PCA–RVM	PCA–RVM–NAR	MLR	SVR	MLP	NARX
PM_2.5_	0.551	0.969	0.978	0.908	0.933	0.907	0.931
PM_10_	− 1.076	0.947	0.972	0.810	0.938	0.827	0.909
CO	− 0.929	0.929	0.970	0.506	0.872	0.708	0.895
NO_2_	− 1.333	0.927	0.957	0.532	0.899	0.752	0.890
SO_2_	− 0.726	0.960	0.980	0.413	0.958	0.786	0.935
O_3_	0.094	0.967	0.981	0.800	0.945	0.864	0.932

**Table 5:** The MAE of self-built point and various air quality prediction models, in which national control point is used as comparison object.

Input variable	Self-built point	PCA–RVM	PCA–RVM–NAR	MLR	SVR	MLP	NARX
PM_2.5_	18.181	4.032	3.430	7.042	5.821	7.763	6.070
PM_10_	50.151	6.958	4.877	13.689	7.080	13.184	9.218
CO	0.549	0.089	0.058	0.263	0.110	0.237	0.100
NO_2_	29.838	4.189	3.144	12.641	4.658	9.991	4.924
SO_2_	12.867	2.090	1.459	10.206	2.116	7.246	2.684
O_3_	36.63	5.702	4.266	16.582	7.647	14.396	7.948

**Table 6:** The MAPE of self-built point and various air quality prediction models, in which national control point is used as comparison object.

Input variable	Self-built point	PCA–RVM	PCA–RVM–NAR	MLR	SVR	MLP	NARX
PM_2.5_	0.447	0.104	0.083	0.166	0.133	0.185	0.151
PM_10_	0.887	0.118	0.086	0.221	0.107	0.210	0.147
CO	0.478	0.097	0.058	0.319	0.112	0.283	0.096
NO_2_	2.129	0.173	0.130	0.639	0.170	0.471	0.1816
SO_2_	0.685	0.134	0.090	0.741	0.131	0.530	0.161
O_3_	4.322	0.294	0.290	1.261	0.373	1.002	0.428

## Conclusions

Air quality is related to the quality of human life^3,4^. The main pollutants affecting air quality are PM_2.5_, PM_10_, CO, NO_2_, SO_2_ and O_3_. Real-time monitoring of pollutant concentrations is of great help for the government and relevant departments to take corresponding measures to pollution sources. The development of miniature air quality detectors is very helpful for human beings to monitor air quality in real time and grid. However, due to various reasons, the measurement accuracy of the miniature air quality detector needs to be improved. The PCA–RVM–NAR model proposed in this study successfully improved the measurement accuracy of the miniature air quality detector by 77.8–93.9%. In addition, the PCA–RVM–NAR model performs very well on both the training set and the test set, indicating that it has a strong generalization ability. It uses a total of 4135 sets of data, and the data of four seasons are covered in the model, which also shows that the model has good stability. However, air quality is affected by many factors. The PCA–RVM–NAR model does not consider other external factors when it is established. Future work can try to introduce more external factors to improve the accuracy of the model. In addition, the climate in different regions is different, and the suitability of the model in other regions also needs further verification.

## Data Availability

The data that support the findings of this study are available from the corresponding author B.L. upon reasonable request.
