# Author Correction: Calibration of miniature air quality detector monitoring data with PCA–RVM–NAR combination model

**DOI:** 10.1038/s41598-025-88487-2

**Published:** 2025-02-04

**Authors:** Bing Liu, Yirui Zhang

**Affiliations:** 1 Public Foundational Courses Department, Nanjing Vocational University of Industry Technology, Nanjing, 210023 China; 2 School of Intelligent Manufacturing, Sanmenxia Polytechnic, Sanmenxia, 472000 China

Correction to: *Scientific Reports* 10.1038/s41598-022-13531-4, published online 04 June 2022

This Article contains errors in the caption of Figure 1:

Comparison of daily average data of six types of pollutants at national control point (NCP) and self-built point (SBP). Figures are generated using Matlab (Version R2016a, https://www.mathworks.com/) [Software].

should read:

Comparison of daily average data of six types of pollutants at national control point (NCP) and self-built point (SBP). Figures are generated using Matlab (Version R2016a, https://www.mathworks.com/) [Software]. Adapted from [22].

[22] Liu, B., Zhao, Q., Jin, Y. *et al.* Application of combined model of stepwise regression analysis and artificial neural network in data calibration of miniature air quality detector. *Sci Rep* 11, 3247 (2021). 10.1038/s41598-021-82871-4

